# High salinity conveys thermotolerance in the coral model Aiptasia

**DOI:** 10.1242/bio.028878

**Published:** 2017-11-24

**Authors:** Hagen M. Gegner, Maren Ziegler, Nils Rädecker, Carol Buitrago-López, Manuel Aranda, Christian R. Voolstra

**Affiliations:** Red Sea Research Center, Division of Biological and Environmental Science and Engineering (BESE), King Abdullah University of Science and Technology (KAUST), Thuwal 23955-6900, Saudi Arabia

**Keywords:** Coral reefs, Climate change, Symbiosis, Thermotolerance, Resilience, *Symbiodinium*, Bleaching, Heat stress

## Abstract

The endosymbiosis between dinoflagellate algae of the genus *Symbiodinium* and stony corals provides the foundation of coral reef ecosystems. Coral bleaching, the expulsion of endosymbionts from the coral host tissue as a consequence of heat or light stress, poses a threat to reef ecosystem functioning on a global scale. Hence, a better understanding of the factors contributing to heat stress susceptibility and tolerance is needed. In this regard, some of the most thermotolerant corals live in particularly saline habitats, but possible effects of high salinity on thermotolerance in corals are anecdotal. Here we test the hypothesis that high salinity may lead to increased thermotolerance. We conducted a heat stress experiment at low, intermediate, and high salinities using a set of host-endosymbiont combinations of the coral model Aiptasia. As expected, all host-endosymbiont combinations showed reduced photosynthetic efficiency and endosymbiont loss during heat stress, but the severity of bleaching was significantly reduced with increasing salinities for one of the host-endosymbiont combinations. Our results show that higher salinities can convey increased thermotolerance in Aiptasia, although this effect seems to be dependent on the particular host strain and/or associated symbiont type. This finding may help explain the extraordinarily high thermotolerance of corals in high salinity environments, such as the Red Sea and the Persian/Arabian Gulf, and provides novel insight regarding factors that contribute to thermotolerance. Since our results are based on a salinity effect in symbiotic sea anemones, it remains to be determined whether this salinity effect can also be observed in stony corals.

## Abstract (precis)

**Summary:** High salinity can be a factor contributing to increased cnidarian thermal tolerance, as indicated by reduced algal endosymbiont loss and reduced photosynthetic impairment in the sea anemone Aiptasia.

## INTRODUCTION

Coral reefs are declining globally due to the direct and indirect effects of climate change (Albright et al., 2016; Anthony et al., 2008; Hoegh-Guldberg, 1999; Hughes et al., 2017a). In particular, ocean warming has eroded the functional basis of these ecosystems, by disrupting the endosymbiosis between corals and photosynthetic algae of the genus *Symbiodinium* (Wild et al., 2011). Elevated sea surface temperatures (SSTs) have caused and continue to cause coral bleaching, that is the loss of *Symbiodinium* as evidenced by the visible whitening of the coral host, on global scales, resulting in the loss of coral cover and destruction of reef ecosystems (Hoegh-Guldberg, 1999; Hoegh-Guldberg and Smith, 1989; Hughes et al., 2017b). While the mechanisms of coral bleaching are still not completely understood (Baird et al., 2009; Pogoreutz et al., 2017; Weis, 2008), the production and accumulation of reactive oxygen species (ROS) and associated oxidative stress is likely playing a major role in heat-induced coral bleaching (Dubinsky and Stambler, 2011; Lesser, 1997).

In comparison to the well-documented detrimental effects of elevated SSTs, much less is known about other environmental drivers and their effect on coral and reef ecosystem functioning. In this context, salinity is commonly regarded as a structuring factor of marine ecosystems, influencing the distribution of plankton, mollusks, fish, and corals (Barletta, 2004; Hoegh-Guldberg, 1999; Rosenberg et al., 1992). Further, salinity is predicted to change as a result of an intensification of the global water cycle driven by climate change (Durack et al., 2012).

For corals, the majority of studies report detrimental effects of hyposaline and hypersaline stress as shown by decreased photosynthetic performance, bleaching, and an increase of coral mortality (Ferrier-Pagès et al., 1999; Gardner et al., 2016; Kerswell and Jones, 2003; Porter et al., 1999). However, corals thrive in naturally highly saline environments, such as the Red Sea or the Persian/Arabian Gulf (Glynn and Wellington, 1983; Riegl et al., 2012). In addition, these environments are characterized by periodically high SSTs highlighting a remarkably high thermal tolerance of corals under these conditions (Coles and Jokiel, 1978; D'Angelo et al., 2015; Howells et al., 2016). Previous studies attributed this temperature tolerance to associations with specialized algal partners (Howells et al., 2016; Hume et al., 2016) or adaptation to extreme temperature and saline environments (D'Angelo et al., 2015), but a direct influence of increased salinity to thermotolerance was not considered.

Here we tested the compelling hypothesis that high salinities increase thermotolerance in the coral model Aiptasia (*sensu Exaiptasia pallida*) (Baumgarten et al., 2015; Grajales et al., 2016; Weis et al., 2008). To do this, we investigated the effect of three different salinities on two Aiptasia strains (H2 and CC7) associated with their native endosymbionts (*Symbiodinium* type B1, strain SSB01, and *Symbiodinium* type A4, strain SSA01, respectively) during a heat stress experiment.

## RESULTS

### Higher salinities reduce bleaching during heat stress in Aiptasia H2 associated with SSB01

**Fig. 1.**
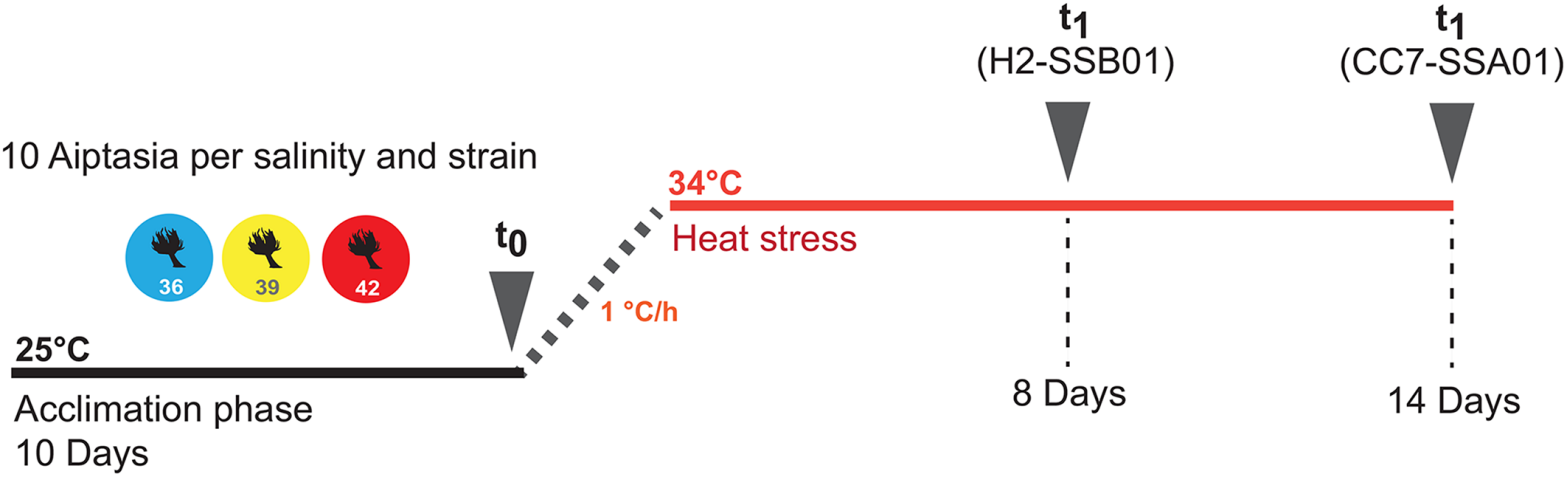
**Experimental design and sampling scheme.** Overview of the acclimation phase and heat stress treatment of the two host-symbiont combinations at three salinities (36, 39, and 42). Sampling points are indicated with t_0_ and t_1_.

**Fig. 2.**
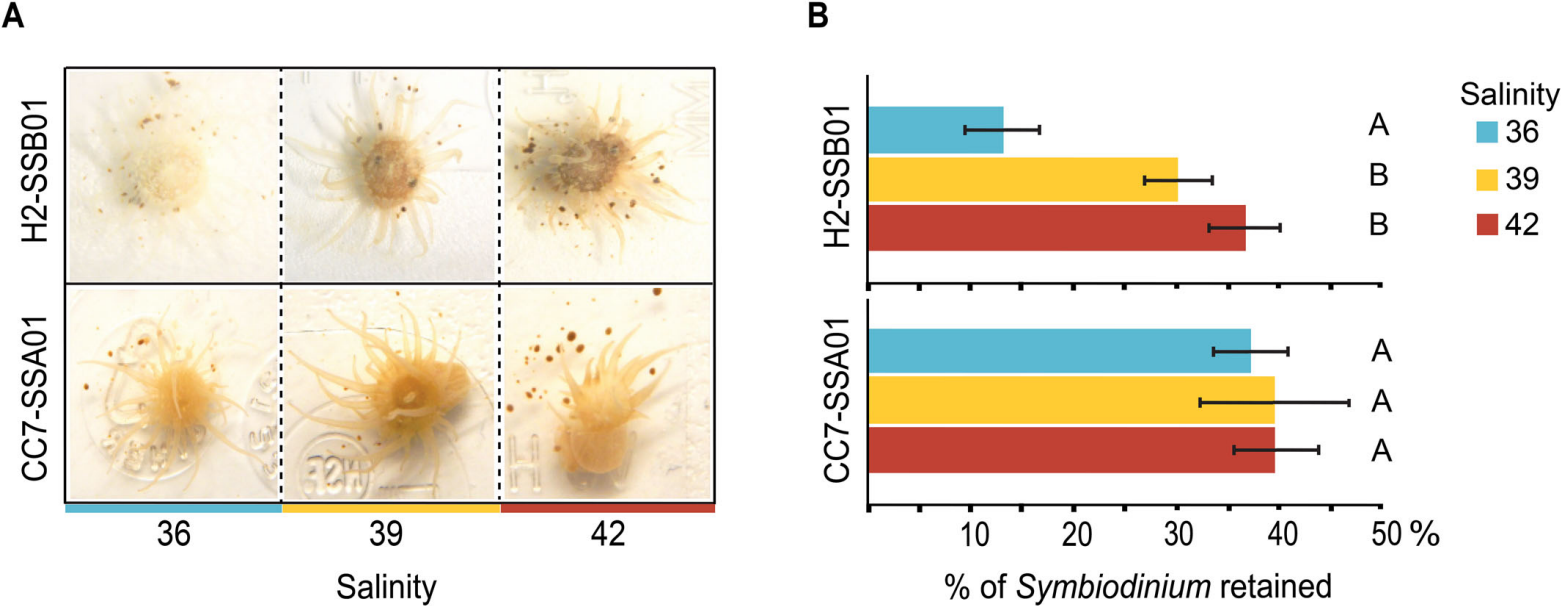
**Effect of different salinities on heat-induced bleaching in the coral model Aiptasia.** (A) Aiptasia H2 associated with *Symbiodinium* SSB01 showed reduced bleaching at increased salinities under heat stress. This pattern was not apparent in Aiptasia CC7 associated with SSA01. (B) Accompanied by the reduced bleaching at increased salinities, Aiptasia H2-SSB01 retained about three times more endosymbionts at increased salinities under heat stress. By comparison, Aiptasia CC7-SSA01 retained equal proportions of endosymbionts under heat stress irrespective of salinity levels. Percentages reported are in relation to *Symbiodinium* counts before the start of the experiment (t_0_). Color key indicates salinity: blue (36), yellow (39), red (42). Data are shown as means±s.e. Different letters indicate significant differences between groups (Tukey's HSD post hoc, *P*<0.05).

To test a possible effect of salinity on thermotolerance and bleaching in the sea anemone Aiptasia, we conducted a long-term heat stress experiment using Aiptasia of the host-symbiont combinations H2-SSB01 and CC7-SSA01 (Figs 1 and 2; Fig. S1). We measured *Symbiodinium* counts at low (36), intermediate (39), and high (42) salinities at the beginning of the experiment (t_0_) and after anemones were either visually bleached or the photosynthetic apparatus was impaired (t_1_) (see Materials and Methods) (Datasets S1 and S2).

At the end of the heat stress experiment, differential effects of salinity on thermotolerance of different host-endosymbiont combinations were evident visually: H2-SSB01 anemones were clearly bleached (translucent) at a low salinity, while clearly pigmented at intermediate and high salinities (Fig. 2). In contrast, CC7-SSA01 displayed a consistently higher pigmentation (as apparent by a uniform brown coloration), regardless of salinity (Fig. 2). Notably, long-term rearing (16 days) of Aiptasia H2-SSB01 and CC7-SSA01 under different salinities at ambient temperature (25°C) did not affect symbiont densities [H2-SSB01 X^2^_(15, 2)_=1.46, *P*>0.1; CC7-SSA01 X^2^_(12, 2)_=2.9231, *P*>0.1] (Fig. S2).

Visual differences were corroborated by changes in endosymbiont densities during the experiment. Both host-endosymbiont combinations exhibited significant loss of *Symbiodinium* during the heat stress experiment at all salinity levels (Fig. 2; Fig. S3, Table S1). However, the severity of bleaching (i.e. loss of *Symbiodinium*) of H2-SSB01 showed a highly significant interaction effect with salinity over time over the course of the heat stress experiment: at intermediate (39) and high (42) salinities significantly more *Symbiodinium* were retained (30.5% and 37.2%, respectively) in comparison to the retention at low (36) salinity (13.6% retention) [X^2^_(30, 2)_=15.88, *P*<0.001]. Conversely, we did not find a significant interaction of salinity over time for CC7-SSA01, since *Symbiodinium* counts did not significantly differ between salinities during heat stress [X^2^_(29, 2)_=0.20, *P*=0.903]. Rather, CC7-SSA01 retained a similar number of *Symbiodinium* cells, irrespective of salinity: 37.4%, 39.7%, and 39.9% of *Symbiodinium* cells were retained at salinities of 36, 39, and 42, respectively (Fig. 2).

Further, we found no significant differences in *Symbiodinium* density for H2-SSB01 between any salinity for t_0_, but significant differences for intermediate and high salinities in comparison to low salinities for t_1_ (Table S2). Also, endosymbiont densities were highly significantly different between t_0_ and t_1_ (Table S2). For CC7-SSA01, no significant differences in symbiont densities across salinities for either time point were found. However, densities between t_0_ and t_1_ were significantly different, denoting the overall loss of *Symbiodinium* (i.e. bleaching) as a result of heat stress.

### Higher salinities reduce photosynthetic impairment during heat stress in Aiptasia H2 associated with SSB01

**Fig. 3.**
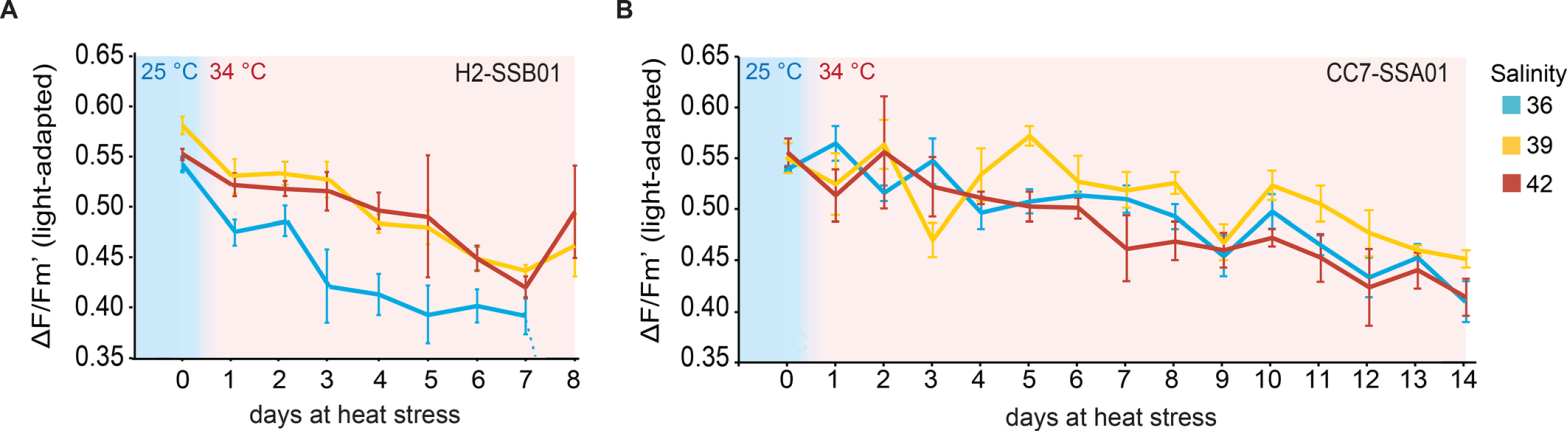
**Effect of different salinities on photosynthetic efficiency during heat stress in the coral model Aiptasia.** Day 0 marks the end of a 10-day acclimation phase at 25°C, after which the temperature was increased to 34°C. Color key indicates salinity: blue (36), yellow (39), red (42). (A) Aiptasia H2 associated with *Symbiodinium* SSB01 showed significantly reduced impairment of photosynthetic efficiencies at increased salinities under heat stress. The heat stress treatment was concluded on day 8 due to substantial bleaching and the fluorescent signal being too low to be measured by Pulse Amplitude Modulated (PAM) fluorometry at a salinity of 36. (B) Photosynthetic efficiency of Aiptasia CC7 associated with *Symbiodinium* SSA01 was significantly less impaired at a salinity of 39 in comparison to the salinities of 36 and 42. Heat stress continued until day 14, when the photosynthetic efficiency for one of the measurements dropped below 0.4. Data are shown as mean±s.e.

We assessed photosynthetic efficiency of H2-SSB01 and CC7-SSA01 at the different salinities during the heat stress experiment (Fig. 3; Table S3, Datasets S3 and S4). Before the start of the heat stress, all host-symbiont combinations displayed common photosynthetic efficiencies (ΔF/Fm’) of around 0.52-0.61 at ambient temperature (25°C), regardless of salinity.

As expected, and in line with an impairment of photosynthesis under heat stress (Lesser, 1996; Warner et al., 1999), we found declining photosynthetic efficiencies for H2-SSB01 and CC7-SSA01 over time at all salinities. Accordingly, we found highly significant differences over time for both host-symbiont combinations [H2-SSB01 X^2^_(30, 2)_=40.93, *P*<0.001; CC7-SSA01 X^2^_(29, 2)_=105.68, *P*<0.001], but also between salinities [H2-SSB01 X^2^_(30, 2)_=51.80, *P*<0.001; CC7-SSA01 X^2^_(29, 2)_=20.11, *P*<0.001]. In addition to an overall decrease of photosynthetic efficiencies over time, this suggests that different salinities exert differential effects on photosynthetic efficiency under heat. In particular, H2-SSB01 was significantly more impaired at low salinity (36) than at intermediate (39) and high (42) salinities (Fig. 3). This was further corroborated by significant differences in photosynthetic efficiencies between the low salinity in comparison to intermediate and high salinities, but not between the latter two (Table S4). On day 8 of the long-term heat-stress experiment, H2-SSB01 at a salinity of 36 was substantially bleached and the fluorescent signal was too low to be measured by a Pulse Amplitude Modulated (PAM) fluorometer (see Materials and Methods, Fig. 3). For CC7-SSA01, by contrast, at both low and high salinities, photosynthetic efficiency was significantly more impaired than at the intermediate salinity (Fig. 3; Table S4), arguing that the highest photosynthetic efficiency was retained at the intermediate salinity. In comparison to H2-SSB01, heat stress for CC7-SSA01 continued until day 14, when the photosynthetic efficiency for one of the measurements dropped below 0.4 (see Materials and Methods, Fig. 3).

## DISCUSSION

Coral reefs worldwide are threatened by the effects of climate change, in particular by ocean warming, as reflected by the most recent mass bleaching of corals (Hughes et al., 2017b). Despite the projected increase and severity of recurrent mass bleaching events, we have a comparatively limited understanding as to the detailed underlying mechanisms and environmental influences that cause bleaching, or how bleaching might be mitigated (e.g. Pogoreutz et al., 2017; Ziegler et al., 2017). Prompted by the notion that the world's most thermotolerant corals also live in the most saline water bodies of the world, and individual studies reporting on high tolerance of corals under high salinity (Coles and Jokiel, 1978; D'Angelo et al., 2015; Howells et al., 2016; Hume et al., 2015), we tested whether increased/high salinity affects thermotolerance using the Aiptasia model system.

Our results show that conditions of high salinity can reduce the severity of bleaching in Aiptasia, as reflected in higher symbiont densities and higher photosynthetic efficiency during heat stress under high salinity conditions. Importantly, this effect was only evident in Aiptasia H2-SSB01, but not in CC7-SSA01. Of note, the host-symbiont combination CC7-SSA01 seemed overall more thermotolerant, as indicated by retaining a similar percentage of symbionts and slower impairment of PSII across salinities. This does not necessarily imply that salinity does not affect thermotolerance in CC7-SSA01. Rather, the effects of salinity-conveyed thermotolerance may be dwarfed by other buffering and regulatory mechanisms by the host and endosymbiont. In this regard, it will be interesting to determine the contribution of the associated *Symbiodinium* type (and any potential contribution of genetic variability within *Symbiodinium* types) to a given host. Aiptasia will provide a powerful platform to address these questions, due to its ability to associate with different symbiont types (Voolstra, 2013; Wolfowicz et al., 2016; Xiang et al., 2013). Future work should also establish whether the observed salinity conveyed thermotolerance of the Aiptasia-*Symbiodinium* symbiosis is directly transferable to other symbiotic cnidarian systems, such as scleractinian corals. At present, we cannot disentangle the individual contribution of the host and endosymbiont to thermotolerance in this study. Nonetheless, our results confirm that increased/high salinity can convey thermotolerance concomitant with reduced bleaching at least for some host-endosymbiont combinations.

Indications for the influence of salinity on coral thermal tolerance date back more than 30 years, but were only observed rather anecdotally. For instance, Coles and Jokiel (1978) found that an increase in salinity (40) above ambient seawater levels (35) led to an increase in resistance to thermal stress. Porter et al. (1999) tested the single and combined effects of temperature and salinity on the coral *Orbicella annularis* in the Florida Keys. The authors found a short-term mitigating effect of high salinity (40) on elevated temperatures (33°C). In contrast to the temperature mitigating effects of high salinity treatments, hyposaline conditions were linked to bleaching and reduction in photosynthetic efficiency of *Symbiodinium* (Kerswell and Jones, 2003). Although it remains to be shown in how far results obtained from Aiptasia hold true for corals, these studies argue that corals may show increased thermotolerance at increased salinities. In this regard, our results do not account for the contribution of host genetic variability, since we used clonal Aiptasia strains. At present, it therefore remains to be determined to what extent host genetic variability may influence the observed salinity effect. A repetition of the experiment with sea anemone individuals from natural populations would allow assessing a potential role of host genetic variability. Importantly, our results already suggest that the observed salinity effect is not universally applicable. Thus, it will be interesting to determine whether our findings can be extrapolated to diverse coral populations and different coral species at large.

The underlying mechanisms that could potentially convey increased thermotolerance in corals at higher salinities are unknown at present. Very recent work studying osmoadaptation in *Symbiodinium* and corals, however, may begin to provide an answer. Ochsenkühn et al. (2017) showed that the osmolyte floridoside is present at high levels in corals and *Symbiodinium* under high salinity conditions, and may serve a dual function as a compatible organic osmolyte and a reactive oxygen species scavenger. As such, floridoside adjusts osmotic pressure and at the same time counters oxidative stress produced as a consequence of salinity and heat stress, thereby contributing to stress resilience. In this regard, it is interesting to note that a study by D'Angelo et al. (2015) found that corals from the Persian/Arabian Gulf show strong local adaptation, not only to high temperatures, but also to the exceptionally high salinity of their habitat. Importantly, the authors could show that the superior heat tolerance is lost when these corals are exposed to reduced salinity levels. An existing relationship between salinity and stress resilience could also explain the higher heat tolerance of corals in the northern Red Sea (Osman et al., 2017) and the Gulf of Aqaba (Fine et al., 2013), in comparison to their central and southern Red Sea counterparts, given the higher salinity in the northern Red Sea. The comparison to other systems reveals that studies from plants grown at high salinities have repeatedly shown an increased temperature tolerance (Lu et al., 2003; Rivero et al., 2014). Interestingly, Rivero et al. (2014) attributed the higher temperature tolerance at high salinities in tomato to an increased production of osmolytes with secondary ROS-scavenging abilities, such as glycine, betaine, or trehalose. These osmolytes therefore serve a dual function: first, they alter and adjust osmotic concentrations, and secondly, they act as antioxidant molecules that can counter the increased ROS produced as a result of high salinity and high temperature (Bose et al., 2014; Hasegawa et al., 2000; Mittler, 2002; Murata et al., 2007). Thus, plants and corals might show the same increased temperature tolerance under high salinity because of similarity in the underlying mechanism. Besides ROS-scavenging abilities of certain osmolytes, it was shown that betaine and trehalose can act as a ‘chemical chaperones’ regulating the activity of molecular chaperones (Diamant et al., 2001). Encouraged by these findings, experiments are currently underway to assess osmolyte production and levels in symbiotic Aiptasia and corals under conditions of high salinity and temperature stress.

### Conclusions

Using the coral model Aiptasia, in this study we show that higher salinities can lead to increased thermotolerance accompanied by less bleaching in a cnidarian-dinoflagellate symbiosis. This salinity effect, however, is not universal, but seems to be dependent on the host, symbiont, or both. At this point, it remains to be determined whether such a salinity effect increases the thermotolerance of stony corals in high salinity environments and whether all coral species respond to it. In this regard, it will be important to better understand the contribution of genetic variation of host and symbiont as well as the role that distinct symbiont types play in salinity-conveyed thermotolerance. Future studies should address the underlying mechanism(s) of salinity-induced thermotolerance in corals from naturally saline regions, such as the Red Sea and Persian/Arabian Gulf, and the implications this may have for corals under altered environmental conditions on a global scale.

## MATERIALS AND METHODS

### Aiptasia rearing, experimental setup, and sample processing

Anemones of the clonal Aiptasia strains H2 (Xiang et al., 2013) and CC7 (Sunagawa et al., 2009) were kept at a 12 h:12 h light/dark cycle at 30-40 μmol m^−2^ s^−1^ at 25°C in small tanks (225 ml) in an incubator (I-36LLVL, Percival Scientific Inc., USA) and fed twice weekly with freshly hatched *Artemia* (brine shrimp larvae). Water was exchanged 24 h after each feeding. We used two strains (representing two genotypes) of two genetically distinct Aiptasia lineages (Thornhill et al., 2013). These two lineages associate with different dominant *Symbiodinium* types and differ in their degree of symbiont diversity and specificity (Thornhill et al., 2013). H2 anemones associated with their native endosymbionts *Symbiodinium* type B1 [strain SSB01, species *Symbiodinium minutum* (Baumgarten et al., 2015; Xiang et al., 2013)], referred to as H2-SSB01, and CC7 anemones associated with their native endosymbionts *Symbiodinium* type A4 [strain SSA01, species *S. linucheae* (Bieri et al., 2016)], referred to as CC7-SSA01.

Animals of both host-endosymbiont combinations were acclimated for 10 days at 25°C to the three experimental salinities (36, low salinity; 39, intermediate salinity; and 42, high salinity) (Fig. 1). For this, 60 animals (30 per host-endosymbiont combination) were distributed over 12 tanks of 225 ml volume (2 tanks per salinity) and feeding was ceased (experimental setup: 5 animals per tank, *2 tanks, *3 salinities, *2 host-symbiont combinations=60 anemones). Different salinities were achieved by diluting autoclaved seawater (obtained from the Red Sea) with ddH_2_0 to a salinity of 36 and subsequent adjustment to experimental salinities with NaCl. After the acclimation phase, five anemones were randomly selected from each salinity treatment for each host-endosymbiont combination (*n*=30 anemones) and each transferred into a single cryotube (time point t_0_). Anemones in cryotubes were immediately shock frozen in liquid nitrogen and stored at −80°C until further processing. The remaining five anemones of each host-endosymbiont combination at each of the three salinities (*n*=30) were subjected to a heat stress experiment. Temperature was ramped from 25°C to 34°C over the course of 10 h (1°C h^−1^ increment) and remained at this level during the course of the heat stress experiment (for CC7-SSA01, 1 anemone at salinity 42 was lost during water exchange). The experiment was terminated for each host-endosymbiont combination, when at least one of the following conditions was met: (1) Aiptasia anemones appeared completely bleached visually, i.e. translucent, or (2) the photosynthetic efficiency dropped below 0.4 (see Results, Fig. 3). Following these criteria, the long-term heat stress experiment for H2-SSB01 was terminated after 8 days and for CC7-SSA01 after 14 days, respectively (Fig. 1). Replicate anemones were each transferred into single cryotubes and immediately shock frozen in liquid nitrogen and stored at −80°C until further processing (time point t_1_). During the course of both experiments, salinity levels were monitored twice a day with a refractometer (Aqua Medic GmbH, Germany). Tanks were cleaned every three days and water was exchanged. Further, to rule out a direct effect of salinity on symbiont density in Aiptasia, we conducted a control experiment in the absence of a heat stress treatment (12 animals per strain and salinity) over the length of 16 days. Samples were processed as described above.

### *Symbiodinium* cell counts and normalization

Frozen anemones (see preceding section, Materials and Methods) were thawed on ice and homogenized for 9 s in 400 µl of sterile saline water (salinity of 36) using a MicroDisTec homogenizer 125 (ThermoFisher Scientific) following the protocol by Krediet et al. (2015). *Symbiodinium* cell counts (three technical replicates per sample) were obtained via flow cytometry (Guava EasyCyte HT, Millipore, USA) using 25 μl of homogenate diluted in 225 μl of 0.1% SDS on 96-well round-bottom plates (Corning Life Sciences, NY, USA) with automatic mixing of each well for 7 s at high speed before quantification. InCyte v2.2 software (Millipore, USA) was used for discrimination of *Symbiodinium* cells from anemone host cells and debris using a combination of side scatter and chlorophyll fluorescence (Krediet et al., 2015). For the control experiment, *Symbiodinium* cell counts were processed in the same way, but measured by flow cytometry using the BD LSRFortess cell analyser (BD Biosciences). For all measurements, technical error rates were <1% as determined by internal standards. *Symbiodinium* cell counts were normalized to total protein content (heat stress experiment) or total host protein (control experiment) of the corresponding anemone homogenates (see preceding section, Materials and Methods) using the Pierce BCA assay (ThermoFisher Scientific) according to manufacturer's instructions. Retained *Symbiodinium* cells (t_1_) were calculated in relation to *Symbiodinium* cell counts before the start of the experiment (t_0_) for each respective salinity.

### Photographic documentation and photosynthetic efficiency

Differences in tissue coloration of symbiotic anemones indicating endosymbiont loss were recorded at the beginning and end of the heat stress experiment for H2-SSB01 and CC7-SSA01 using a Nikon Coolpix AW 130. Light-adapted photosynthetic efficiencies (ΔF/Fm’) of photosystem II (PSII) for each anemone were measured daily for the duration of the long-term heat stress experiment using a diving Pulse Amplitude Modulated (PAM) fluorometer (Walz, Germany).

### Statistical analysis

All statistical analyses were conducted in R v3.3.1 (R Core Team, 2016). For the heat stress experiment, the effects of salinity (36, 39, 42) and time (t_0_ and t_1_) on symbiont density and photosynthetic efficiency were tested using generalized linear models (GLMs) for H2-SSB01 and CC7-SSA01, respectively. Models were fitted to the data using gamma distribution and the best-fitting link function. All models started as two-factorial models accounting for interactive as well as additive effects of salinity and time. This was followed by a step-wise deletion of insignificant response parameters based on the Akaike information criterion (AIC). This resulted in two-factorial interactive models of salinity and time with an ‘identity’ link function in the case of symbiont density, and two-factorial non-interactive models of salinity and time with an ‘identity’ link function for photosynthetic efficiency data for H2-SSB01 and CC7-SSA01, respectively. Following model optimization, comparisons between individual salinities and time points were conducted using the Tukey post hoc test, as implemented in the ‘multcomp’ package (Hothorn et al., 2008). For the control experiment, a non-parametric Kruskal–Wallis test was used to test whether salinity had an effect on *Symbiodinium* cell densities of the two host combinations (H2-SSB01 and CC7-SSA01) in the absence of heat stress.

## Supplementary Material

Supplementary information
