# Supplementary material for: High salinity conveys thermotolerance in the coral model Aiptasia
Source: Biol Open. 2017 Nov 24;6(12):1943–8. doi: 10.1242/bio.028878 (PMC5769654; doi:10.1242/bio.028878)
Supplement: Supplementary information [file biolopen-6-028878-s1.pdf]

## Supplementary Information

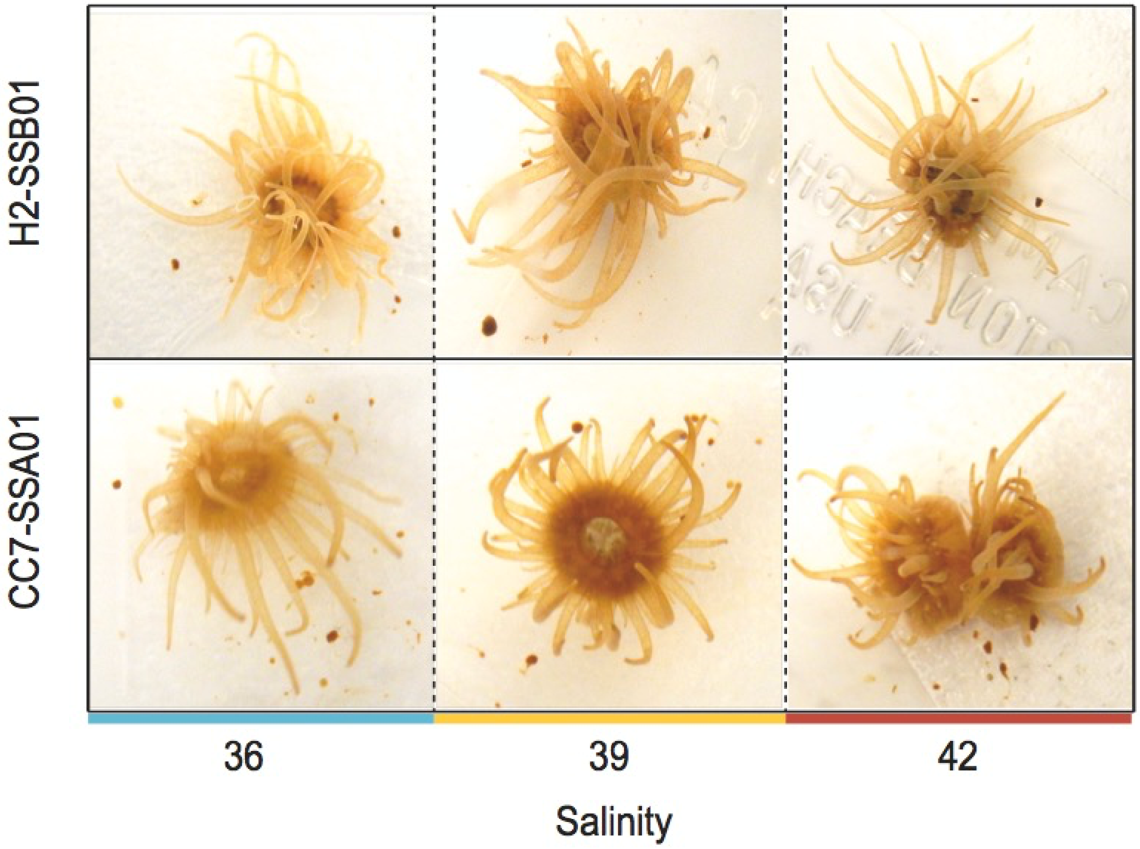


**Figure S1.** Aiptasia H2-SSB01 and CC7-SSA01 in low (36), intermediate (39) and high (42) salinity before start of the long-term heat stress experiment ($t_0$).

Biology Open • Supplementary information

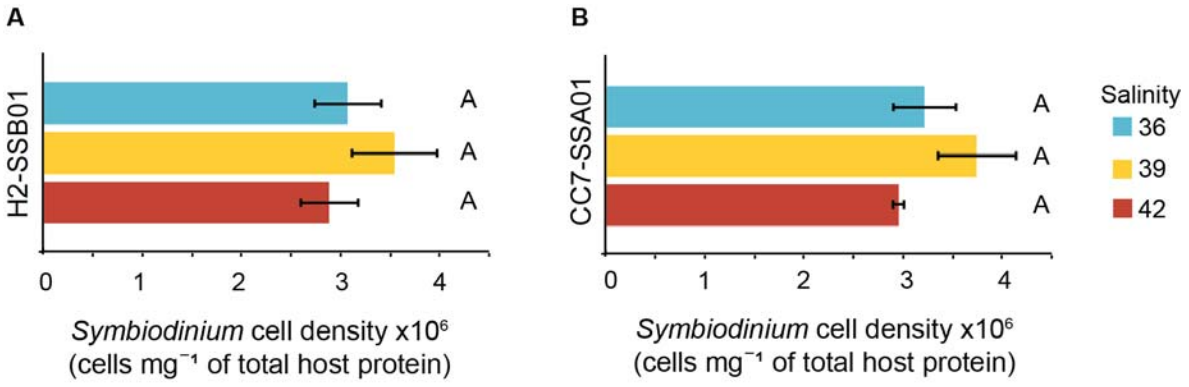


**Figure S2. *Symbiodinium* densities after long-term rearing (16 days) of Aiptasia under different salinities at ambient temperature (25 °C). (A)** H2-SSB01. **(B)** CC7-SSA01. Total host protein was used for normalization. Color key indicates salinity: blue (36), yellow (39), red (42). Data are shown as means ± SE. Letters indicate no significant differences between groups (Kruskal-Wallis, H2-SSB01 $P > 0.1$; CC7-SSA01 $P > 0.1$).

Biology Open • Supplementary information

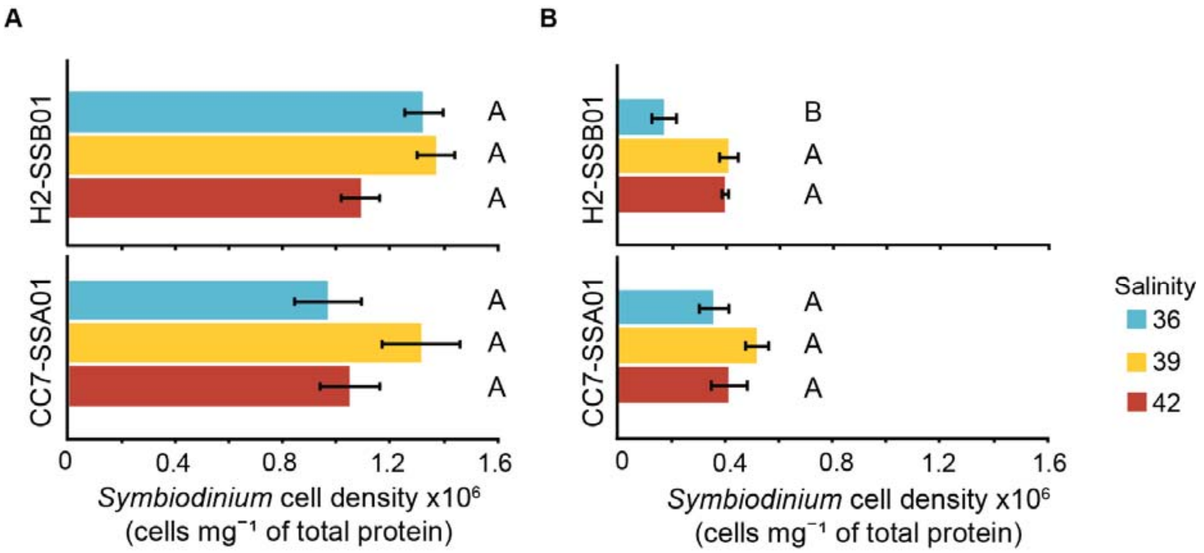


**Figure S3. *Symbiodinium* cell densities of Aiptasia. (A)** Before the heat stress experiment ($t_0$). (**B**) After the heat stress experiment ($t_1$). Total protein was used for normalization. Color key indicates salinity: blue (36), yellow (39), red (42). Data are shown as means ± SE. Different letters indicate significant differences between groups (Tukey's HSD post-hoc, $P < 0.05$).

**Table S1. Analysis of two-factorial generalized linear models (GLMs) on *Symbiodinium* cell counts with salinity and time as individual and interactive explanatory variables for Aiptasia (A) H2-SSB01 and (A) CC7-SSA01.** All models are based on gamma distribution and best fitting link function. Bold values indicate $P < 0.05$.

| **(A) H2-SSB01** | **$X^2$** | **Df** | ***P*** |
|---|---|---|---|
| Time | 145.77 | 1 | **< 0.001** |
| Salinity | 8.88 | 2 | **0.012** |
| Time:Salinity | 15.88 | 2 | **< 0.001** |

| **(B) CC7-SSA01** | **$X^2$** | **Df** | ***P*** |
|---|---|---|---|
| Time | 80.67 | 1 | **< 0.001** |
| Salinity | 7.56 | 2 | **0.023** |
| Time:Salinity | 0.20 | 2 | 0.903 |

Biology Open • Supplementary information

**Table S2. Tukey's HSD post-hoc comparison of *Symbiodinium* cell counts between time points and salinities for Aiptasia (A) H2-SSB01 and (B) CC7-SSA01.** Pairwise comparisons are denoted as Salinity (36,39.42).Timepoint (T0,T1). Bold values indicate $P < 0.05$; SE = standard error.

**(A) H2-SSB01**

| **Pairwise comparison** | **Estimated** | **SE** | **z value** | ***P*** |
|---|---|---|---|---|
| 36.T0 - 39.T0 | 0.03 | 0.19 | 0.18 | 0.999 |
| 36.T0 - 42.T0 | -0.19 | 0.188 | -1.03 | 0.908 |
| 39.T0 - 42.T0 | -0.23 | 0.188 | -1.21 | 0.833025 |
| 36.T1 - 36.T0 | -2.00 | 0.188 | -10.60 | < **0.001** |
| 39.T1 - 36.T0 | -1.15 | 0.188 | -6.13 | < **0.001** |
| 42.T1 - 36.T0 | -1.18 | 0.188 | -6.28 | < **0.001** |
| 36.T1 - 39.T0 | -2.03 | 0.188 | -10.78 | < **0.001** |
| 39.T1 - 39.T0 | -1.19 | 0.188 | -6.31 | < **0.001** |
| 42.T1 - 39.T0 | -1.22 | 0.188 | -6.46 | < **0.001** |
| 36.T1 - 42.T0 | -1.80 | 0.188 | -9.57 | < **0.001** |
| 39.T1 - 42.T0 | -0.96 | 0.188 | -5.10 | < **0.001** |
| 42.T1 - 42.T0 | -0.99 | 0.188 | -5.25 | < **0.001** |
| 36.T1 - 39.T1 | 0.84 | 0.188 | 4.48 | < **0.001** |
| 36.T1 - 42.T1 | 0.81 | 0.188 | 4.32 | < **0.001** |
| 39.T1 - 42.T1 | -0.03 | 0.188 | -0.15 | 1.000 |

**(B) CC7-SSA01**

| **Pairwise comparison** | **Estimated** | **SE** | **z value** | ***P*** |
|---|---|---|---|---|
| 36.T0 - 39.T0 | 0.30 | 0.174 | 1.74 | 0.503 |
| 36.T0 - 42.T0 | 0.08 | 0.174 | 0.46 | 0.998 |
| 39.T0 - 42.T0 | -0.22 | 0.174 | -1.29 | 0.792 |
| 36.T1 - 36.T0 | -0.98 | 0.174 | -5.64 | < **0.001** |
| 39.T1 - 36.T0 | -0.62 | 0.174 | -3.56 | **0.005** |
| 42.T1 - 36.T0 | -0.84 | 0.174 | -4.54 | < **0.001** |
| 36.T1 - 39.T0 | -1.29 | 0.174 | -7.39 | < **0.001** |
| 39.T1 - 39.T0 | -0.92 | 0.174 | -5.30 | < **0.001** |
| 42.T1 - 39.T0 | -1.14 | 0.185 | -6.18 | < **0.001** |
| 36.T1 - 42.T0 | -1.06 | 0.174 | -6.10 | < **0.001** |
| 39.T1 - 42.T0 | -0.70 | 0.174 | -4.01 | < **0.001** |
| 42.T1 - 42.T0 | -0.92 | 0.185 | -4.97 | < **0.001** |
| 36.T1 - 39.T1 | 0.36 | 0.174 | 2.09 | 0.294 |
| 36.T1 - 42.T1 | 0.15 | 0.185 | 0.79 | 0.970 |
| 39.T1 - 42.T1 | -0.22 | 0.185 | -1.18 | 0.846 |

Biology Open • Supplementary information

**Table S3. Analysis of two-factorial generalized linear model (GLM) for photosynthetic efficiency analysis with salinity and time as individual explanatory variables for Aiptasia (A) H2-SSB01 and (B) CC7-SSA01.** All models based on gamma distribution and best fitting link function. Bold values indicate $P < 0.05$.

| **(A) H2-SSB01** | **$X^2$** | **Df** | ***P*** |
|---|---|---|---|
| Time | 40.93 | 1 | < **0.001** |
| Salinity | 51.80 | 2 | < **0.001** |

| **(B) CC7-SSA01** | **$X^2$** | **Df** | ***P*** |
|---|---|---|---|
| Time | 105.68 | 1 | < **0.001** |
| Salinity | 20.11 | 2 | < **0.001** |

**Table S4. Tukey's HSD post-hoc comparison of photosynthetic efficiencies between salinities over the course of the long-term heat stress experiment for Aiptasia (A) H2-SSB01 and (B) CC7-SSA01**. Pairwise comparisons are denoted as Salinity(36,39,42) – Salinity(36,39,42). Bold values indicate *P* < 0.05; SE = standard error.

**(A) H2-SSB01**

| Pairwise comparison | Estimated | SE | z value | *P* |
|---|---|---|---|---|
| 39 - 36 | 0.07 | 0.011 | 6.26 | < **0.001** |
| 42 - 36 | 0.07 | 0.011 | 6.26 | < **0.001** |
| 42 - 39 | 0.00 | 0.012 | 0.15 | 0.987 |

**(B) CC7-SSA01**

| Pairwise comparison | Estimated | SE | z value | *P* |
|---|---|---|---|---|
| 39 - 36 | 0.02 | 0.008 | 3.00 | **0.009** |
| 42 - 36 | -0.01 | 0.008 | -1.53 | 0.276 |
| 42 - 39 | -0.03 | 0.008 | -4.38 | < **0.001** |

Biology Open • Supplementary information

## Supplementary Datasets

**Dataset S1. *Symbiodinium* cell counts based on flow cytometry and normalized to total protein for Aiptasia H2-SSB01.** Time points denoted as T0 (before long-term heat stress experiment) and T1 (after long-term heat stress experiment); salinity indicated by numbers (36, 39 and 42). Biological replicates numbered 1-5, e.g. T0.36.1.

Click here to Download Data Set S1

**Dataset S2. *Symbiodinium* cell counts based on flow cytometry and normalized to total protein for Aiptasia CC7-SSA01.** Time points denoted as T0 (before long-term heat stress experiment) and T1 (after long-term heat stress experiment); salinity indicated by numbers (36, 39 and 42). Biological replicates numbered 1-5, e.g. T0.36.1.

Click here to Download Data Set S2

**Dataset S3. Photosynthetic efficiency over the course of the long-term heat stress experiment for Aiptasia H2-SSB01.** Repeated measures denote biological replicates (i.e. different Aiptasia anemones).

Click here to Download Data Set S3

**Dataset S4. Photosynthetic efficiency over the course of the long-term heat stress experiment for Aiptasia CC7-SSA01.** Repeated measures denote biological replicates (i.e. different Aiptasia anemones).

Click here to Download Data Set S4
